# Positive Effect of Cognitive Reserve on Episodic Memory, Executive and Attentional Functions Taking Into Account Amyloid-Beta, Tau, and Apolipoprotein E Status

**DOI:** 10.3389/fnagi.2021.666181

**Published:** 2021-05-28

**Authors:** Justinas Narbutas, Daphne Chylinski, Maxime Van Egroo, Mohamed Ali Bahri, Ekaterina Koshmanova, Gabriel Besson, Vincenzo Muto, Christina Schmidt, André Luxen, Evelyne Balteau, Christophe Phillips, Pierre Maquet, Eric Salmon, Gilles Vandewalle, Christine Bastin, Fabienne Collette

**Affiliations:** ^1^GIGA Institute, Cyclotron Research Centre In Vivo Imaging, University of Liège, Liège, Belgium; ^2^Psychology and Neuroscience of Cognition Research Unit, Faculty of Psychology and Educational Sciences, University of Liège, Liège, Belgium; ^3^Department of Neurology, CHU de Liège, Liège, Belgium

**Keywords:** cognitive performance, cognitive reserve, allostatic load, AD biomarkers, APOE, midlife

## Abstract

Studies exploring the simultaneous influence of several physiological and environmental factors on domain-specific cognition in late middle-age remain scarce. Therefore, our objective was to determine the respective contribution of modifiable risk/protective factors (cognitive reserve and allostatic load) on specific cognitive domains (episodic memory, executive functions, and attention), taking into account non-modifiable factors [sex, age, and genetic risk for Alzheimer’s disease (AD)] and AD-related biomarker amount (amyloid-beta and tau/neuroinflammation) in a healthy late-middle-aged population. One hundred and one healthy participants (59.4 ± 5 years; 68 women) were evaluated for episodic memory, executive and attentional functioning *via* neuropsychological test battery. Cognitive reserve was determined by the National Adult Reading Test. The allostatic load consisted of measures of lipid metabolism and sympathetic nervous system functioning. The amyloid-beta level was assessed using positron emission tomography in all participants, whereas tau/neuroinflammation positron emission tomography scans and apolipoprotein E genotype were available for 58 participants. Higher cognitive reserve was the main correlate of better cognitive performance across all domains. Moreover, age was negatively associated with attentional functioning, whereas sex was a significant predictor for episodic memory, with women having better performance than men. Finally, our results did not show clear significant associations between performance over any cognitive domain and apolipoprotein E genotype and AD biomarkers. This suggests that domain-specific cognition in late healthy midlife is mainly determined by a combination of modifiable (cognitive reserve) and non-modifiable factors (sex and age) rather than by AD biomarkers and genetic risk for AD.

## Introduction

The constant increase in life expectancy is associated with an increase in age-related physical and cognitive dysfunctions (WHO, 2011). Age-related cognitive changes include a broad spectrum of clinical categories, including cognitively normal aging, subjective cognitive decline, and mild cognitive impairment, and dementia stages. It is now clear that age-related brain changes associated with Alzheimer’s disease (AD) begin at least as early as in late middle age (Beason-Held et al., 2013; Jack and Holtzman, 2013; Ritchie et al., 2015; Coupé et al., 2019). For example, deposits of amyloid-beta (Aβ) and tau protein, two main biomarkers of AD pathophysiology, are observed decades before the first clinical signs of cognitive decline (Jack and Holtzman, 2013).

The presence of these biomarkers does not, however, mean that people will necessarily develop clinical AD; they are only indicative of an increased risk for developing the disease (Dumurgier et al., 2017; Roe et al., 2018). This implies that the presence of risk factors can potentially be counterbalanced by protective factors, which may explain the diversity in decline rates and trajectories of cognitive aging (Nyberg et al., 2012; Reuter-Lorenz and Park, 2014; Cabeza et al., 2018). Besides Aβ and tau proteins (Betthauser et al., 2019), several physiological and psychological environmental protective and risk factors were proposed to explain this variability (Norton et al., 2014; Livingston et al., 2017), such as cognitive reserve (Cabeza et al., 2018; Stern et al., 2018), allostatic load (Karlamangla et al., 2014), and genetics, particularly with ε4 polymorphism in apolipoprotein E (APOE) gene (Greenwood et al., 2014).

As current research in cognitive aging shifts to primary and secondary prevention, it is important to identify predictors of cognitive performance as early as possible. Late midlife is, therefore, an interesting time window, sometimes characterized by first manifestations of AD pathophysiology and subtle cognitive changes or cognitive complaints. Moreover, several chronic diseases or lifestyle choices such as hypertension, diabetes, obesity, sedentary, and smoking habits are related to subsequent brain pathology and cognitive decline in later life (Debette et al., 2011; Livingston et al., 2020). In addition, the factors affecting cognition in late midlife differ from factors that are important in older age (McFall et al., 2019). Therefore, a better understanding of risk and protective factors for cognition in late midlife would provide valuable insight for lifestyle changes and/or interventions.

Cognitive reserve refers to adaptability (i.e., efficiency, capacity, and flexibility) of cognitive processes that help counteract physiological changes associated with brain aging, pathology, or insult (Stern et al., 2018). Cognitive reserve helps to maintain normal cognitive performance in the presence of pathology through the recruitment of alternative brain networks, altered brain metabolism (Bastin et al., 2012), or alternative cognitive strategies. The influence of cognitive reserve factors on cognition in normal aging observed in cross-sectional studies is grounded on the positive association between cognitive efficiency, which refers to optimal performance on a cognitive task (Hoffman and Schraw, 2010), and (a) higher level of education and intelligence (Fratiglioni and Wang, 2007; Meng and D’Arcy, 2012; Osone et al., 2015; Bright et al., 2018) for reviews), (b) employment complexity and autonomy (Bosma et al., 2003; Ansiau et al., 2005; Baldivia et al., 2008; Then et al., 2014 for reviews), and (c) physical activity, engagement in cognitively demanding leisure activities, and/or sustained social interactions (Wang et al., 2012 for a review). These factors also seem to influence cognitive decline in longitudinal studies (Schooler et al., 1999; Bosma et al., 2003; Manly et al., 2003; Andel et al., 2014; Hughes et al., 2018), but the effects are more mixed than in cross-sectional designs (see Pettigrew and Soldan, 2019 for a review and discussion). They also delay pathological cognitive decline, with a later onset of AD in individuals with higher reserve (Stern et al., 2018). Importantly, these cognitive reserve factors may have compensatory effects already in middle-aged people, as shown for global cognition and visual abilities (Ferreira et al., 2017) and other cognitive functions, including episodic memory, executive and attentional processes, but excluding processing speed (De Frias and Dixon, 2014; Hughes et al., 2018).

In contrast, the strains put on physiology and health have a negative influence on cognitive aging. Several studies emphasized a link between cardiovascular functioning and cognition already in midlife (Dahle et al., 2009; Gupta et al., 2015; Zeki Al Hazzouri et al., 2017), whereas lipid and glucose metabolism, inflammation, cortisol level, and sympathetic nervous system functioning were associated with early cognitive decline (Karlamangla et al., 2005; Wright et al., 2006; Dahle et al., 2009; Ownby, 2010; Sartori et al., 2012; Ma et al., 2017; Narbutas et al., 2019). The allostatic load was proposed as a comprehensive index gathering the physiological stressors (Karlamangla et al., 2002) that are negatively associated with episodic memory performance and executive functioning in middle-aged and older adults (Karlamangla et al., 2014).

Also, ε4 polymorphism in the APOE gene is an established genetic risk factor for late-onset AD (Corder et al., 1993). However, its link with cognitive performance in healthy late middle-aged individuals remains inconsistent in cross-sectional studies, with positive, negative, and null associations reported with cognition, especially for memory performance (for a review, see Salvato, 2015).

Moreover, there is a broad discussion about the implication of both pathologic AD proteins (Aβ and tau) in cognitive performance in late midlife. Whereas some studies found a link between positron emission tomography (PET) Aβ accumulation and episodic memory decline in midlife (Hedden et al., 2013; Schultz et al., 2015; Clark et al., 2016, 2019; Farrell et al., 2017), other studies did not find any significant relationship (Johnson et al., 2014; Oh et al., 2014; Song et al., 2015; Mielke et al., 2016). Furthermore, greater PET Aβ deposition in middle age was associated with lower vocabulary level (Farrell et al., 2017) and decreased attentional and executive functioning in some (Doherty et al., 2015; Clark et al., 2016; Mielke et al., 2016) but not all studies (Johnson et al., 2014; Hollands et al., 2015; Lim et al., 2015; Pietrzak et al., 2015; Song et al., 2015). A recent study combining both Aβ and second-generation tau PET tracers in a group of late-middle-aged healthy participants demonstrated a combined detrimental influence on cognition of both proteins: participants with both elevated Aβ and tau levels experienced three times faster cognitive decline in comparison with those having just one or no elevated biomarkers (Betthauser et al., 2019). In addition, each biomarker may be associated with specific aspects of cognition, with tau level associated with verbal episodic memory and Aβ deposit with executive performance (Terrera et al., 2020).

Studies exploring the simultaneous influence of several physiological and psychological risk and protective factors on cognition in late middle age remain scarce. Our previous cross-sectional study (Narbutas et al., 2019) in late middle-aged individuals demonstrated that several categories of modifiable lifestyle factors, i.e., cognitive reserve and allostatic load, and particularly some subfactors, i.e., crystallized intelligence, sympathetic nervous system functioning, and lipid metabolism, may explain the variability in cognitive performance measured on a global composite score, which is sensitive to early cognitive change (Papp et al., 2017). In a similar vein, in a sample of healthy older adults aged from 53 to 85 years old, stable memory performance was associated with higher education, lower depressive symptoms, better living status, normal body mass index (BMI), normal heart rate, and more social activities, whereas declining memory performance was associated with fewer novel cognitive activities, reduced grip strength, abnormal heart rate, and poorer gait (McFall et al., 2019). Furthermore, McFall et al. (2019) reported that the association of these factors with memory status was more marked in the young-old group (mean age: 64.1 years).

In the continuity of these studies, our objective was to determine the respective contribution of modifiable risk/protective factors (allostatic load and cognitive reserve) on specific cognitive domains (episodic memory, executive functions, and attention), taking into account non-modifiable factors (sex, age, and APOE status) and brain AD-related biomarker level (Aβ and tau) in a healthy late-middle-aged population with a negative status for AD pathology. When initiating the study, [18F]THK5351 was a promising tau radiolabel. However, as detailed in the method section, [18F]THK5351 was later found to have an important unspecific binding to the neuroinflammation element (Chiotis et al., 2018). Although unintended, results with [18F]THK5351 will therefore reflect a combined burden of tau and neuroinflammation. This remains of interest as neuroinflammation is gaining increased attention as a potential mediator of cognitive impairment in aging (Kumar, 2018). With the present dataset, the authors predicted that protective factors (higher cognitive reserve and female sex) would be related to better performance in domain-specific cognition, whereas adverse factors (worse allostatic load, older age, APOE ε4 carrier status, higher Aβ, and tau/neuroinflammation burden) would be related to worse performance. We further anticipated that the influence of protective and adverse factors should be visible only for episodic memory and executive function (Hohman et al., 2017; McFall et al., 2019; Veldsman et al., 2020). As tau and APOE status were available only in a subsample of participants, analyses including these factors will be presented as exploratory.

## Materials and Methods

### Participants

One hundred and one healthy late middle-aged (50–69 years old) French-speaking community-dwelling men and women (Table 1; *N* = 101; 68 women [67.3%]) enrolled in a study designed to identify biomarkers and lifestyle factors associated with normal cognitive aging in the context of preclinical dementia [the Cognitive Fitness in Aging study, see Narbutas et al. (2019)]. No participants reported any recent history of neurological or psychiatric disease or were taking medication affecting the central nervous system. All had normal or corrected-to-normal vision and hearing. Other exclusion criteria were sleep apnea/hypopnea index ≥ 15/h, assessed during an in-lab night of sleep under polysomnography, BMI < 18 and > 30 kg/m^2^, smoking, illicit drug consumption, excessive consumption of caffeine (>4 cups/day) or alcohol (>14 units/week), diabetes, and shift-work over the past 6 months. Participants with high levels of depression and anxiety as assessed by the Beck Depression Inventory (Beck et al., 1961) and by the 21-item self-rated Beck Anxiety Inventory (Beck et al., 1988), respectively, were excluded (i.e., score > 17), as well as participants with ongoing pharmacological treatment of depression or anxiety. Participants with treated (>6 months) hypertension and hypothyroidism were included. All participants showed normal performance on the Mattis Dementia Rating Scale (Mattis, 1976) (i.e., score > 130). The experimental procedures were approved by the local Ethics Committee of the Faculty of Medicine (University of Liège) and were in accordance with the Code of Ethics of the World Medical Association (Declaration of Helsinki) for experiments involving humans. All participants gave their signed informed consent before the experiment, they acknowledge that they cannot be identified *via* the paper, were fully anonymized, and received a financial compensation.

**TABLE 1:** Descriptive statistics of demographical data and cognitive outcome (*n* = 101).

	**Mean**	***SD***	**Minimum**	**Maximum**
**Demographical data**				
Age, *years*	59.44	5.29	50.00	69.00
Sex, *female, n (%)*	68 (67.3%)			
Ethnic status, *Caucasian, n (%)*	101 (100%)			
Educational level:				
Primary school	0 (0%)			
Secondary school	26 (25.7%)			
Bachelor degree	37 (36.6%)			
Master degree	31 (30.7%)			
PhD or higher	7 (6.9%)			
Beck Depression Inventory	5.22	4.44	0	17.00
Beck Anxiety Inventory	2.88	3.19	0	17.00
Mattis Dementia Rating Scale	142.53	1.87	134.00	144.00
Cognitive Difficulties Scale	27.89	19.55	0	95.00
**Episodic memory score**	0	2.17	–8.31	4.40
FCSRT	47.48	6.44	34.00	60.00
Logical memory task, *delayed recall*	12.13	4.03	2.00	22.00
MST, *recognition memory score*	0.79	0.15	–0.06	1.00
**Executive functioning score**	0	3.31	–6.42	8.97
Phonemic Fluency Test, *2 min score*	24.66	7.04	7.00	48.00
Semantic Fluency Test, *2 min score*	33.82	7.00	20.00	60.00
Digit Span Task, *score for inverse order*	6.61	2.18	2.00	13.00
TMT*, *RT (s) for part B*	68.67	20.33	32.00	127.00
N-Back, *d′ for 3-back variant*	1.18	0.76	–0.26	3.76
SCWT*, *interference index*	0.10	0.06	–0.02	0.30
**Attentional functioning score**	0	3.09	–7.35	6.37
DSST, *total 2 min score*	72.57	12.57	39.00	99.00
TMT*, *RT (s) for part A*	31.70	8.67	18.00	60.00
N-Back, *d′ for 1-back variant*	4.70	0.92	1.94	5.76
D2 Test of Attention, *GZ-F score*	401.73	71.87	185.00	610.00
CRTT*, *RT (ms) for non-identical items*	694.61	118.88	459.94	1090.31

*SD, standard deviation; RT, reaction time; d′, sensitivity index; FCSRT, Free and Cued Selective Reminding Test (sum of all free immediate recalls + free delayed recall); MST, Mnemonic Similarity Task; TMT, Trail Making Test; SCWT, Stroop Color and Word Test; DSST, Digit Symbol Substitution Test; CRTT, Choice Reaction Time Task (see “Materials and Methods” section for references of tests). ^∗^Score inverted for further composite score calculation; thus, higher composite scores reflect better performance*.

### Experimental Design

In this multimodal cross-sectional study, we assessed domain-specific cognitive performance, i.e., episodic memory and executive and attentional functioning, as well as cognitive reserve, allostatic load, and APOE status. We assessed Aβ cortical level and phosphorylated tau accumulation in regions affected at Braak stages 1 and 2 of AD using PET. All participants also underwent quantitative multi-parametric magnetic resonance imaging (MRI) acquisitions for subsequent preprocessing of PET data. PET tau protein measurement and APOE status were available only for 58 participants. All mandatory laboratory health and safety procedures have been complied with in the course of conducting the experimental work reported in the present article.

### Neuropsychological Examination

The neuropsychological evaluation consisted of a battery of cognitive tasks assessing short-term and episodic memory and attentional and executive functions. The episodic memory composite score included: Free and Cued Selective Reminding Test (sum of all free immediate recalls + free delayed recall) (Grober et al., 2009), Logical Memory Task (delayed recall) (Wechsler and Stone, 1987), and Mnemonic Similarity Task (recognition memory score) (Stark et al., 2015). The executive functioning composite score comprised: Phonemic Fluency Test (2 min score) and Semantic Fluency Test (2 min score for an animal category) (Cardebat et al., 1990), Backward Digit Span Task (total score for inverse order) (Wechsler, 1997), Trail Making Test (inverted reaction time in seconds for part B) (Bowie and Harvey, 2006), N-Back Visual Task (*d*′ for three-back variant) (Kirchner, 1958), and a computerized version of the Stroop Color and Word Test (inverted interference index) (Stroop, 1935). The attentional functioning composite score included: Digit Symbol Substitution Test (total 2 min score) (Wechsler, 1997), Trail Making Test (inverted reaction time in seconds for part A) (Bowie and Harvey, 2006), N-Back Visual Task (*d*′ for one-back variant) (Kirchner, 1958), D2 Test of Attention (GZ-F score) (Brickenkamp, 1966), and Choice Reaction Time Task (inverted reaction time in milliseconds for non-identical items) (Zimmermann and Fimm, 2002). We computed a composite *z*-score for each cognitive domain based on the sum of *z*-scores on domain-related tasks, with higher scores reflecting better performance. Neuropsychological evaluation was performed during two sessions of approximately 75 min, which included additional neuropsychological tasks that are not considered in the present analyses.

### Cognitive Reserve

A measure of the cognitive reserve was based on previous results (Narbutas et al., 2019) and consisted of the total score at the National Adult Reading Test French version (fNART) (Nelson, 1982), also considered as a measure of crystallized intelligence. The fNART is considered a good proxy of cognitive reserve (Manly et al., 2003; Starr and Lonie, 2008; Osone et al., 2015).

### Allostatic Load

Measures of allostatic load were based on previous methodology (Narbutas et al., 2019) and consisted of lipid metabolism and sympathetic nervous system functioning. The lipid metabolism index was calculated based on measures of BMI, waist–hip ratio, and blood measures of low-density lipoprotein cholesterol, high-density lipoprotein cholesterol, and triglycerides. The sympathetic nervous system functioning index was calculated based on the following urinary measures: 24-h excretion of adrenaline and 24-h excretion of noradrenaline. Both adrenaline and noradrenaline excretions were also corrected for serum creatinine level to adjust for lean body mass (Karlamangla et al., 2014). Final measures of lipid metabolism and sympathetic nervous system functioning were standardized (i.e., *z*-scored), and higher values mean higher allostatic load.

### Genotype

APOE genotyping was performed using blood sample DNA extraction. Common single-nucleotide polymorphisms were assessed using Infinium OmniExpress-24 BeadChip (Illumina, San Diego, CA, United States) based on human genome build hg19 (GRCh37). Genotype imputation was performed using the ‘‘Sanger Imputation Server’’^1^ by choosing Haplotype Reference Consortium (release 1.1) (Auton et al., 2015) as reference panel and the pre-phasing algorithm Eagle2 (Loh et al., 2016). APOE variants were determined by rs7412 and rs429358 single-nucleotide polymorphisms. Participants were classified into ε4 carries (heterozygous and homozygous) and non-carriers.

### Magnetic Resonance Imaging (MRI)

Quantitative multi-parametric MRI acquisition was performed on a 3-T MR scanner (Siemens MAGNETOM Prisma, Siemens Healthineers, Erlangen, Germany). Quantitative maps were obtained by combining images using different parameters sensitive to distinct tissue properties. Multiparameter mapping was based on a multi-echo three-dimensional fast low angle shot at 1-mm isotropic resolution (Weiskopf and Helms, 2008). This included three datasets with T1-, proton density-, and magnetization transfer (MT)-weighted contrasts imposed by choice of the flip angle (FA = 6° for proton density and MT and 21° for T1) and the application of an additional off-resonance Gaussian-shaped radiofrequency pulse for the MT-weighted acquisition. MRI multiparameter maps were processed with the hMRI toolbox^2^ (Tabelow et al., 2019) and SPM12 (Welcome Trust Centre for Neuroimaging, London, United Kingdom) to obtain notably a quantitative MT map as well as segmented images (gray matter, white matter, and cerebrospinal fluid), normalized to the standard MNI space using unified segmentation (Ashburner and Friston, 2005).

### Positron Emission Tomography PET

PET acquisition was performed on an ECAT EXACT + HR scanner (Siemens, Erlangen, Germany) with [18F]Flutemetamol (*N* = 97) or [18F]Florbetapir (*N* = 3) radiotracers to assess Aβ burden and with [18F]THK-5351 with the intention to measures tau burden. Although first-generation tau protein radiotracers, including [18F]THK5351, have been widely studied and proved to have high affinity and selectivity *in vitro* (Lemoine et al., 2017), an important off-binding (∼50%) to monoamine oxidase B (MAO-B) *in vivo*, particularly over the basal ganglia (Chiotis et al., 2018), was reported. A recent comparative analysis of first- and second-generation radiotracers reported that this off-target binding might be common to all first-generation radiotracers and, to a lesser extent, also to second-generation radiotracers (Murugan et al., 2019). Accordingly, the [18F]THK5351 binding we measured is a combined marking of phosphorylated tau and of neuroinflammatory elements such as reactive astrocytes, which may be triggered in part by tau pathology (Harada et al., 2018).

For all radiotracers, participants received a single dose of the respective radio-ligands in an antecubital vein (target dose approximately 185 MBq). Aβ-PET image acquisitions started 85 min after injection, and four frames of 5 min were obtained. For [18F]THK-5351-PET, a 10-min transmission scan was acquired first, and dynamic image acquisitions started immediately after injection, consisting of 32 frames (with increasing time duration), with a total duration spent in the scanner approximately 100 min. All PET images were reconstructed using a filtered back-projection algorithm, including corrections for measured attenuation, dead time, random events, and scatter using standard software.

**FIGURE 1:**
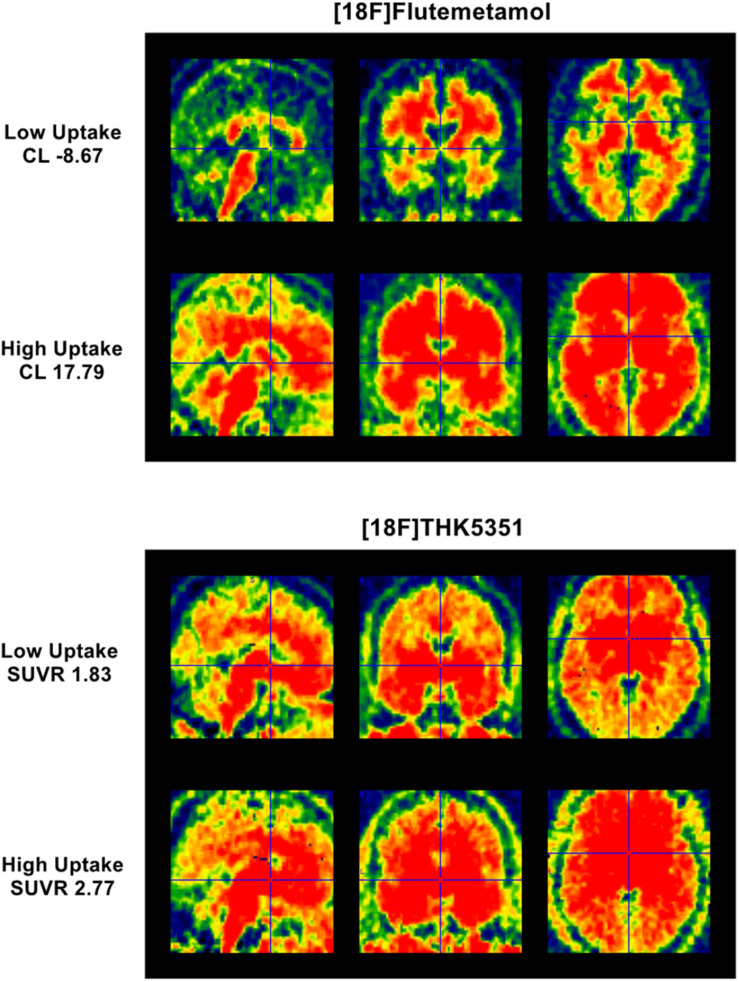
Parametric [18F]Flutemetamol and [18F]THK5351 uptake. **(Top)** panel represents parametric [18F]Flutemetamol binding estimation for subject having lowest Centiloid value (–8.67 CL) and for subject having highest Centiloid value (17.79 CL) over a mask covering mostly neocortical regions. **(Bottom)** panel represents parametric [18F]THK5351 binding estimation for subject having lowest SUVR value (1.83 SUVR) and for subject having highest SUVR value (2.77 SUVR) over a mask covering entorhinal cortex and hippocampus.

Average PET images were created using the 4 [18F]Flutemetamol/[18F]Florbetapir frames and using four frames (40–60 min) for [18F]THK-5351-PET (Lockhart et al., 2016). Averaged PET images were manually reoriented and automatically coregistered to the individual space structural MT map. Flow-field deformation parameters obtained from DARTEL spatial normalization of MT maps were applied to averaged coregistered PET images. A standardized uptake value ratio was calculated using the whole cerebellum as the reference region for Aβ-PET (Klunk et al., 2015) and cerebellum gray matter for [18F]THK-5351-PET (Ishiki et al., 2018). Volumes of interests (VOIs) were determined using automated anatomical labeling atlas, except for brainstem, entorhinal cortex, and basal ganglia VOIs that were defined with WFU PickAtlas 3.0.5b toolbox on MATLAB 2013a (MathWorks Inc., Natick, MA, United States). As Aβ-PET images were obtained using two radiotracers, their standardized uptake value ratio values were scaled to Centiloid units (CL) (Klunk et al., 2015; Battle et al., 2018; Navitsky et al., 2018) (for further details, see Supplementary Material Table 1). Aβ burden was averaged over a large mask covering neocortical regions (frontal, temporal, and parietal cortices, insular cortex, precuneus, and anterior striatum) shown to distinguish AD patients from controls (Klunk et al., 2015) (see Figure 1). [18F]THK-5351-PET burden (see Figure 1) was averaged over regions corresponding to Braak stages 1 and 2 (entorhinal cortex and hippocampus) (Braak and Braak, 1991; Schöll et al., 2016). Among 100 subjects having Aβ-PET scans, five subjects were considered as outliers (>3 SD from the sample mean) and removed before subsequent analyses. For [18F]THK-5351, one subject was considered as an outlier (>3 SD from the sample mean) and excluded from the study sample (final sample = 58).

### Statistical Analyses

We performed statistical analyses with SAS 9.4 for Windows (SAS Institute, Cary, NC, United States). Generalized linear mixed models (GLMM; PROC GLIMMIX) were applied to compute all statistics following the determination of the distribution of dependent variables using “allfitdist” function on MATLAB R2013a (MathWorks Inc., Natick, MA, United States). In all GLMMs, collinearity diagnosis was performed on all predictors using Tolerance (TOL) and Variance Inflation Factors (VIF) as criteria. Degrees of freedom were estimated using Kenward–Roger’s correction. Subject (intercept) effect was included as a random factor. We defined a *p*-value < 0.05 as significant.

All models included the composite score from one cognitive domain (episodic memory, executive functions, and attention) as the dependent variable and included sex and age as covariates. At first, GLMMs evaluated the association between each specific cognitive domain and risk/protective factors (fNART, sympathetic functioning, and lipid metabolism) in separate models. Subsequent models included these factors and Aβ burden on the Centiloid scale. A last exploratory model on a subsample (*N* = 58) included the same factors, APOE, and both AD biomarkers (Aβ, [18F]THK-5351 uptake). Moreover, based on previous findings showing that the interaction between Aβ and tau may be even more important for cognitive performance than their unilateral effect (Hanseeuw et al., 2019), we explored this type of interaction in models having APOE and both AD biomarkers (*N* = 58). Comparison of Bayesian Information Criterion values for models with and without interactive term for Aβ and [18F]THK-5351 uptake showed better explanatory power when interaction effects were not modeled. Consequently, only models without interaction terms were analyzed. Semi-partial R2 (*R*_*sp*_^2^) was reported for each significant effect as described previously (Jaeger et al., 2017), provided that degrees of freedom are estimated using Kenward–Roger’s methods.

For the sake of completeness, we also run these models with outliers included and using more targeted masks for Aβ (posterior cingulate cortex and precuneus) (Insel et al., 2020) and [18F]THK-5351 uptake (entorhinal cortex) (Timmers et al., 2019). Results did not fundamentally amend those obtained in the main analyses and are presented in Appendix Tables A1–A3,B1–B3.

## Results

### Descriptive Statistics

Demographics and cognitive outcome are presented in Table 1, whereas Table 2 gathers raw values of cognitive reserve, allostatic load, Aβ and tau burden, and APOE.

**TABLE 2:** Descriptive statistics of raw values of cognitive reserve, allostatic load, Aβ, tau, and APOE.

	**Mean**	***SD***	**Minimum**	**Maximum**
**Cognitive reserve measure (*n* = 101)**				
fNART	28.89	4.03	13.00	36.00
**Allostatic load measures (*n* = 101)**				
*Lipid metabolism*				
Body mass index, *kg/m^2^*	24.62	2.88	17.85	30.12
Waist-hips ratio, *cm/cm*	1.11	0.15	0.72	1.49
LDL, *mg/dl*	138.15	37.12	45.00	272.00
HDL, *mg/dl* (higher is better)	65.31	17.84	28.00	149.00
Triglycerides, *mg/dl*	109.62	55.35	29.00	339.20
*Sympathetic nervous system functioning*				
Urine adrenaline, μ*g/24 h*	8.29	4.56	2.18	25.47
Urine noradrenaline, μ*g/24 h*	37.49	16.23	6.94	95.89
**Aβ (*n* = 95)**				
CL, centiloid brain mask	0.31	4.57	–8.67	17.79
**THK5351 uptake (*n* = 58)**				
SUVR, Braak 1 and 2 mask	2.27	0.21	1.83	2.77
**APOE (*n* = 58)**				
APOE ε4 carriers	10 (17.2%)			
APOE ε4 non-carriers	48 (82.8%)			

*SD, standard deviation; fNART, National Adult Reading Test French version; LDL, fasting blood low-density lipoprotein cholesterol; HDL, fasting blood high-density lipoprotein cholesterol; APOE, apolipoprotein E; Aβ, amyloid-beta; CL, Centiloid units; SUVR, standardized uptake value ratio (see “Materials and Methods” section for the references of tests and questionnaires).*

### Episodic Memory

#### Effect of Cognitive Reserve and Allostatic Load

**FIGURE 2:**
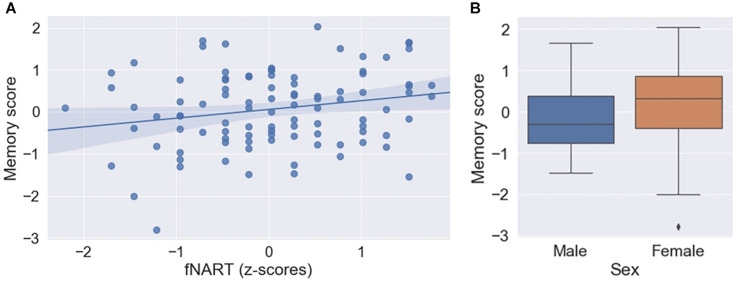
**(A)** Scatter plot displaying association between episodic memory and fNART (*N* = 101). Regression was used for visual display only and not as a substitute for full GLMM statistics. **(B)** Box plot visualizing episodic memory score according to sex (*N* = 101).

Assessment of the association between episodic memory and measures of cognitive reserve and allostatic load showed a significant positive association with the score of the fNART (Table 3A and Figure 2A). Scores of allostatic load were, however, not associated with episodic memory performance. Moreover, sex was also significantly associated with episodic memory, with better performance for women (Figure 2B).

**TABLE 3:** Statistical outcome of GLMM examining associations between episodic memory (dependent variable) and: **(A)** scores of cognitive reserve and allostatic load (*n* = 101); **(B)** scores of cognitive reserve and allostatic load, and Aβ (n = 95); **(C)** scores of cognitive reserve and allostatic load, Aβ, tau, and APOE (*n* = 58).

**Model A** (*n* = 101)	**Estimate ± SE**	***F*-value (df)**	***P***
Sex*	−0.47 ± 0.21	5.05 (1,95)	**0.03** (*R*_*sp*_^2^ = 0.05)
Age	−0.03 ± 0.10	0.12 (1,95)	0.73
fNART	0.31 ± 0.10	10.73 (1,95)	**0.002** (*R*_*sp*_^2^ = **0.10**)
Lipid metabolism	−0.06 ± 0.10	0.39 (1,95)	0.53
Sympathetic functioning	−0.11 ± 0.09	1.32 (1,95)	0.25
**Model B (*n* = 95)**			
Sex*	−0.40 ± 0.22	3.29 (1,88)	0.07
Age	−0.001 ± 0.10	0.0 (1,88)	0.99
fNART	0.30 ± 0.10	8.92 (1,88)	**0.004** (*R*_*sp*_^2^ = 0.09)
Lipid metabolism	−0.07 ± 0.10	0.47 (1,88)	0.50
Sympathetic functioning	−0.10 ± 0.10	0.88 (1,88)	0.35
Aβ	−0.02 ± 0.10	0.05 (1,88)	0.82
**Model C (*n* = 58)**			
Sex*	−0.04 ± 0.30	0.02 (1,49)	0.89
Age	−0.20 ± 0.14	2.12 (1,49)	0.15
fNART	0.24 ± 0.15	2.79 (1,49)	0.10
Lipid metabolism	0.04 ± 0.14	0.08 (1,49)	0.78
Sympathetic functioning	−0.09 ± 0.15	0.34 (1,49)	0.56
Aβ	−0.08 ± 0.15	0.25 (1,49)	0.62
THK5351 uptake	−0.01 ± 0.14	0.01 (1,49)	0.93
APOE	0.13 ± 0.36	0.14 (1,49)	0.71

SE, standard error; df, degrees of freedom; R_*sp*_^2^, Semi-partial R2; fNART, National Adult Reading Test (French version); Aβ, amyloid-beta; APOE, apolipoprotein E. *Sex: 1 = male; 2 = female.

#### Simultaneous Effect of Modifiable and Non-modifiable Factors

Subsequent models included the modifiable factors of cognitive reserve and allostatic load together with non-modifiable factors (Aβ in a first model, *N* = 95; tau and APOE in a second model, *N* = 58). No association between memory performance and Aβ burden was detected beyond its association with fNART values (Table 3B). Similarly, no associations between memory performance and tau burden and APOE genotype were detected in the last model (Table 3C).

Finally, we tentatively ran models including outliers and using other brain region masks for early Aβ/THK5351 uptake. The only observed change is that the positive association observed with the score of the fNART now remains significant (see Appendix Tables A1, B1, respectively, in Supplementary Material Table 2).

### Executive Functions

#### Effect of Cognitive Reserve and Allostatic Load

**FIGURE 3:**
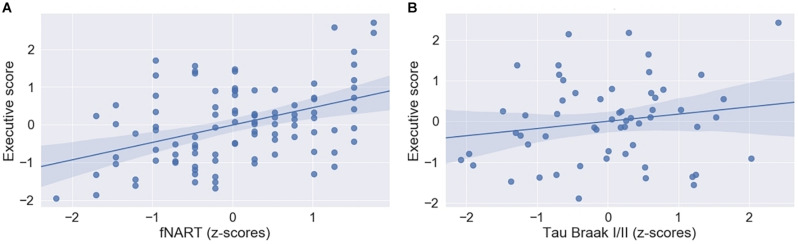
**(A)** Scatter plot displaying association between executive functioning and fNART (*N* = 101). **(B)** Scatter plot displaying association between executive functioning and tau level in Braak regions 1 and 2 (*N* = 58). Regressions were used for visual display only and not as a substitute for full GLMM statistics.

Executive functioning was significantly and positively associated with fNART values (Table 4A and Figure 3A), but it was not the case for measures of allostatic load (Table 4A).

**TABLE 4:** Statistical outcome of GLMM examining associations between executive functions (dependent variable) and: **(A)** scores of cognitive reserve and allostatic load (*n* = 101); **(B)** scores of cognitive reserve and allostatic load, and Aβ (*n* = 95); **(C)** scores of cognitive reserve and allostatic load, Aβ, tau, and APOE (*n* = 58).

**Model A (*n* = 101)**	**Estimate ± *SE***	***F*-value (df)**	***P***
Sex*	0.06 ± 0.20	0.10 (1,95)	0.75
Age	−0.17 ± 0.09	3.30 (1,95)	0.07
fNART	0.46 ± 0.09	25.49 (1,95)	**<0.0001** (*R*_*sp*_^2^ = 0.21)
Lipid metabolism	0.07 ± 0.09	0.60 (1,95)	0.44
Sympathetic functioning	0.12 ± 0.09	1.89 (1,95)	0.17
**Model B (*n* = 95)**			
Sex*	0.04 ± 0.21	0.05 (1,88)	0.83
Age	−0.17 ± 0.10	2.96 (1,88)	0.09
fNART	0.47 ± 0.10	23.73 (1,88)	**<0.0001** (*R*_*sp*_^2^ = **0.21**)
Lipid metabolism	0.07 ± 0.10	0.59 (1,88)	0.45
Sympathetic functioning	0.13 ± 0.09	1.86 (1,88)	0.18
Aβ	0.05 ± 0.10	0.24 (1,88)	0.63
**Model C (*n* = 58)**			
Sex*	−0.17 ± 0.25	0.46 (1,49)	0.50
Age	−0.13 ± 0.12	1.28 (1,49)	0.26
fNART	0.59 ± 0.12	23.09 (1,49)	**<0.0001** (*R*_*sp*_^2^ = **0.32**)
Lipid metabolism	−0.02 ± 0.12	0.02 (1,49)	0.89
Sympathetic functioning	0.06 ± 0.13	0.25 (1,49)	0.62
Aβ	0.12 ± 0.13	0.91 (1,49)	0.35
THK5351 uptake	0.25 ± 0.12	4.49 (1,49)	**0.04** (*R*_*sp*_^2^ =.080.08)
APOE	0.46 ± 0.31	2.24 (1,49)	0.14

*SE, standard error; df, degrees of freedom; R_*sp*_^2^, semi-partial R2; fNART, National Adult Reading Test (French version); Aβ, amyloid-beta; APOE, apolipoprotein E. ^∗^Sex: 1 = male; 2 = female.*

#### Simultaneous Effect of Modifiable and Non-modifiable Factors

Subsequent models included the modifiable factors of cognitive reserve and allostatic load together with non-modifiable factors (Aβ in a first model, *N* = 95; tau and APOE in a second model, *N* = 58). Similarly to episodic memory, no association between Aβ burden and executive performance was detected beyond its association with fNART values (Table 4B). In contrast, there was a significant positive association between executive functioning and tau burden (Table 4C and Figure 3B), whereas the association was not significant with the APOE genotype.

When tentatively running models including outliers and using other brain region masks for early Aβ/THK5351 uptake, the association was not significant anymore with these masks (see Appendix Tables A2, B2, respectively, in Supplementary Material Table 2).

### Attentional Functions

#### Effect of Cognitive Reserve and Allostatic Load

**FIGURE 4:**
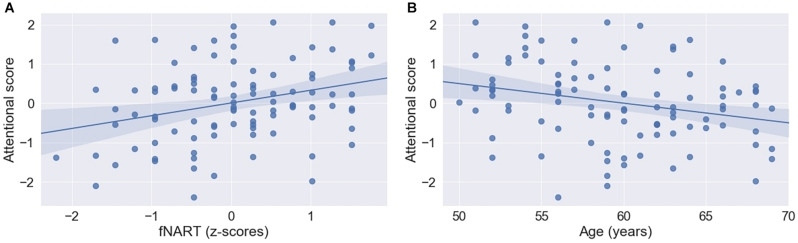
Scatter plots visualizing association between attentional functioning, fNART **(A)**, and age **(B)** (*N* = 101). Regressions were used for visual display only and not as a substitute for full GLMM statistics.

Similar to the other cognitive domains, attentional functioning was significantly and positively associated with fNART values but not with measures of allostatic load (Table 5A and Figure 4A). Moreover, age was significantly negatively associated with attentional functions (Figure 4B).

**TABLE 5:** Statistical outcome of GLMM examining associations between attentional functioning (dependent variable) and: **(A)** scores of cognitive reserve and allostatic load (*n* = 101); **(B)** scores of cognitive reserve and allostatic load, and Aβ (*n* = 95); **(C)** scores of cognitive reserve and allostatic load, Aβ, tau, and APOE (*n* = 58).

**Model A (*n* = 101)**	**Estimate ± *SE***	***F*-value (df)**	***P***
Sex*	0.006 ± 0.20	0.0 (1,95)	0.98
Age	−0.26 ± 0.09	7.78 (1,95)	**0.006** (*R*_*sp*_^2^ = 0.08)
fNART	0.37 ± 0.09	16.15 (1,95)	**0.0001** (*R*_*sp*_^2^ = 0.15)
Lipid metabolism	0.02 ± 0.09	0.06 (1,95)	0.81
Sympathetic functioning	0.16 ± 0.09	3.00 (1,95)	0.09
**Model B (*n* = 95)**			
Sex*	−0.05 ± 0.21	0.05 (1,88)	0.82
Age	−0.22 ± 0.10	5.18 (1,88)	**0.03** (*R*_*sp*_^2^ (0.06)
fNART	350.35 ± 0.10	13.12 (1,88)	**0.0005** (*R*_*sp*_^2^ = **0.13**)
Lipid metabolism	0.04 ± 0.10	0.21 (1,88)	0.65
Sympathetic functioning	0.18 ± 0.10	3.74 (1,88)	0.06
Aβ	−0.07 ± 0.10	0.47 (1,88)	0.49
**Model C** (*n* = 58)			
Sex*	0.002 ± 0.27	0.0 (1,49)	0.995
Age	−0.19 ± 0.13	2.15 (1,49)	0.15
fNART	0.42 ± 0.13	9.87 (1,49)	**0.003** (*R*_*sp*_^2^ = **0.19**)
Lipid metabolism	0.01 ± 0.13	0.01 (1,49)	0.91
Sympathetic functioning	0.19 ± 0.14	1.86 (1,49)	0.18
Aβ	−0.04 ± 0.14	0.07 (1,49)	0.79
THK5351 uptake	0.08 ± 0.13	0.41 (1,49)	0.52
APOE	0.12 ± 0.34	0.12 (1,49)	0.73

*SE, standard error; df, degrees of freedom; R_*sp*_^2^, semi-partial R2; fNART, National Adult Reading Test (French version); Aβ, amyloid-beta; APOE, apolipoprotein E.*

*^∗^Sex: 1 = male; 2 = female*.

#### Simultaneous Effect of Modifiable and Non-mdifiable Factors

When including modifiable factors together with non-modifiable factors (Aβ in a first model, *N* = 95; tau and APOE in a second model, *N* = 58), attentional functioning was not significantly associated with Aβ burden beyond its association with fNART values (Table 5B). As for memory performance, an attentional function also was not significantly associated with tau burden or APOE genotype (Table 5C).

There were no changes in the associations observed with the exploratory models, including outliers and using other brain region masks for early Aβ/THK5351 uptake (see Appendix Tables A3, B3, respectively, in Supplementary Material Table 2).

## Discussion

This study investigated whether performance assessed over episodic memory, executive and attentional domains in healthy late-middle-aged individuals is associated with a combination of risk/protective factors (crystallized intelligence, sympathetic functioning and lipid metabolism, sex, age, and APOE status) as well as with levels of AD-related brain biomarkers (Aβ and THK5351 uptake). Our main finding is that higher crystallized intelligence, a robust proxy of cognitive reserve (Manly et al., 2003; Starr and Lonie, 2008; Osone et al., 2015), is the main correlate of better cognitive performance across all domains. Our results further indicated that age was negatively associated with attentional functioning but not with other cognitive domains, whereas we also detected a significant effect of sex on episodic memory, with women having better performance than men. Finally, our results did not reveal significant associations between performance over any cognitive domain and APOE genotype or AD biomarkers, except for an unanticipated positive link between [18F]THK5351 binding over Braak 1 and 2 stage regions and executive performance, which may be specific to the brain region considered.

### Modifiable Factors (Cognitive Reserve and Allostatic Load)

A positive influence of specific aspects of cognitive reserve on cognition was previously reported in older individuals (Ansiau et al., 2005; Bright et al., 2018; Falck et al., 2018; Ihle et al., 2018), as well as in late midlife (Narbutas et al., 2019). We provide evidence that cognitive reserve (assessed by crystallized intelligence) is positively associated with performance across all three domain-specific cognition with important effect sizes (semi-partial R2 ranging from 0.09 to 0.32 depending on cognitive domain and covariates included in the models). Crystallized intelligence has been shown to be an even better predictor of cognitive efficiency in aging than education (Manly et al., 2003; Osone et al., 2015), and the current study clearly points out the predictive value of crystallized intelligence for cognitive efficiency in late-middle-aged population devoid of significant AD pathology. Nevertheless, the associations between specific cognitive domains and the measure of cognitive reserve (fNART) we observed here may simply mean that fluid intelligence matches quite well with crystallized intelligence in that type of population.

In addition to a significant positive link between cognitive reserve and domain-specific cognition, we also demonstrate that this link remains unaffected by the inclusion of Aβ burden PET marker for executive and attentional functioning, and our results further support that it is also the case for APOE and tau/neuroinflammation status, although based on a subsample only.

Surprisingly, sympathetic functioning and lipid metabolism, two specific measures of allostatic load, were not related to domain-specific cognition in our sample. This is in contrast to previous studies showing that higher allostatic load, a comprehensive index of physiological load related to stress, is related to worse global cognitive performance in late midlife (Narbutas et al., 2019) and both episodic memory and executive functioning in middle-aged and older adults (Karlamangla et al., 2014). It may be that the effect of the allostatic load has a more important manifestation on cognition when the cognitive status is measured using tools sensitive to early cognitive decline (Narbutas et al., 2019). In fact, cognitive composites assessing cognitive status over several specific cognitive domains were proposed as an efficient tool to track the earliest cognitive change (Rentz et al., 2013; Papp et al., 2017).

To sum up, our results are only partially in line with previous findings showing an important effect of modifiable factors promoting late-life cognition, including higher educational level, treatment of cardiovascular diseases, diet, calorie restriction, and exercise, or more generally allostatic load (Karlamangla et al., 2014; Norton et al., 2014; Livingston et al., 2017; Bhatti et al., 2020). We observed an expected positive influence of cognitive reserve by its proxy of crystallized intelligence but no effect of health-related factors. A possible explanation is that the effect of health-related factors on specific cognitive processes could be mediated by other factors not investigated here, such as changes in brain structure and function (Booth et al., 2015; Neth et al., 2020).

### Non-modifiable Factors (Sex, Age, and Apolipoprotein EAPOE)

We detected a significant effect of sex for episodic memory, with women having better performance than men, but only without controlling for AD biomarker status and APOE, which may imply that AD biomarker and APOE are explaining a small and non-significant portion of the variance that allows for sex to emerge as significant. Sex was reported to be an important predictor for stable memory in aging and for cognitive efficiency in late midlife, with women showing less decline on a combined score using four recall measures from two standard episodic memory tasks (McFall et al., 2019) and better performance on the Preclinical Alzheimer Cognitive Composite score (PACC5), a composite measure known to be sensitive to early subtle cognitive changes, possibly leading to dementia (Narbutas et al., 2019). Sex differences were also observed in specific cognitive domains such as episodic memory, executive and attentional functioning, and language in older participants (mean age = 67 years) (McCarrey et al., 2016). It also seems that women have a greater number of factors predicting memory resilience than men (McDermott et al., 2017). For example, a sex difference in cognitive performance may be explained by hormonal differences (Koyama et al., 2016), genetic factors, differences in brain networks, socioeconomic roles, and health choices (Deckers et al., 2018).

We further found that older age was related to worse attentional functioning. This link may be explained by the dependence of general attentional functions, including working memory, on processing speed (Mella et al., 2015), at least for our composite score of attention, which required lots of resources for visual stimuli detection and fast manual responses. Indeed, processing speed is known as the cognitive function, which is affected early in life (i.e., from the third decade) and undergoes a stable decline in cognitive aging (Eckert et al., 2010). In contrast, we did not detect a significant age effect for the executive and memory domains. A recent study that identified modifiable risk factors for episodic memory maintenance and/or decline in healthy individuals also revealed that the influence of those factors differed according to age strata (55–72.4 vs. 72.5–95 years) (McFall et al., 2019). However, age itself was not a robust predictor for memory maintenance or decline in that study. In our sample with a relatively narrow age range (50–69 years), crystallized intelligence seems, therefore, to be associated more strongly with performance in episodic memory and executive functions than age.

Finally, we found that APOE status had no significant impact on domain-specific cognition, which is in line with previous data focusing on episodic memory (Salvato, 2015) and working memory (Greenwood et al., 2014).

### Alzheimer’s Disease AD Biomarkers

Aβ level was not related to domain-specific cognitive measures both when assessing the effect of Aβ alone or when also including THK5153 uptake in a subsample of participants. A similar picture emerges for our exploration of the association between domain-specific cognitive performance and THK5251 uptake except for a surprising positive association with executive performance. The latter results contrast with the scarce previous studies that quite consistently reported that higher global or large neocortical regional tau level was mostly related to worse episodic memory performance, such as list learning, which predominantly relies on the hippocampus (Aschenbrenner et al., 2018; Ossenkoppele et al., 2019; Terrera et al., 2020).

Our surprising finding may arise from the unspecific binding of THK5351 to MAO-B in reactive astrocytes (Lemoine et al., 2017). Neuroinflammation processes involve the activation of glial cells (microglia and astrocytes) and the release of inflammatory mediators (cytokines) (López-Valdés and Martínez-Coria, 2016). The initial function of neuroinflammation is to protect the central nervous system from pathogens by isolating damaged tissue from uninjured areas and cleaning and repairing the extracellular matrix. In contrast, chronic inflammation leads to the degradation of neural tissue and of the blood–brain barrier. Moreover, during normal aging, glial cells become more reactive, which may exacerbate cytokine response and subsequent cognitive decline (Godbout and Johnson, 2009). Neuroinflammation may, in fact, drive part of AD pathology independently of Aβ accumulation (Cunningham et al., 2009; Heppner et al., 2015; López-Valdés and Martínez-Coria, 2016). The link between neuroinflammation and cognition was rarely assessed over specific cognitive domains. Because of the mixed binding of THK5351 to tau and MAO-B, our results do not provide, however, clear insights about this question, but they warrant future research using *in vivo* markers specific to neuroinflammation, such as [11C]PK11195 PET ligand (Kreisl et al., 2020).

The unanticipated link between THK5351 uptake and executive performance may also be due to the binding of the marker to actual tau aggregates. Early tau accumulation begins in the entorhinal cortex and hippocampus (Braak and Braak, 1991) and brainstem nuclei such as the dorsal raphe and locus coeruleus (Uematsu et al., 2018). Therefore, one could speculate that a link between better executive performance and higher tau accumulation in the entorhinal cortex and hippocampus may be explained by compensatory mechanisms in the brain networks encompassing executive functions (e.g., prefrontal cortex), which are activated when tau level increases in the medial temporal lobe (MTL). There is indeed evidence of functional connectivity between the prefrontal cortex and MTL through thalamic pathways (Ketz et al., 2015), and fiber bundles connecting the prefrontal cortex and MTL were reported both in monkeys and humans (Folloni et al., 2019). However, as this link is no more significant when more targeted masks are used in the analyses (see Appendix Table C2), the results require replication, and their interpretation shall be considered tentative.

This raises the question of the brain region considered when assessing the link between AD PET biomarkers and behavior. For Aβ, we have used a VOI covering most neocortical regions and the anterior striatum, as it was reported to be a sensitive mask to distinguish between AD patients and controls (Klunk et al., 2015). For THK5351 uptake, the VOI encompassed Braak stages 1 and 2 regions, which are undergoing initial tauopathy (entorhinal cortex and hippocampus) (Schöll et al., 2016). Smaller VOIs including only regions implicated in specific cognitive domains may be more precise in isolating a link with specific cognitive domains. Indeed, recent evidence prompts that each biomarker is associated with a specific cognitive function that has its regional neural correlates, with a primarily hippocampal task (e.g., verbal episodic memory) being associated with higher levels of tau and a frontal executive task (e.g., coding) related to higher levels of amyloid (Terrera et al., 2020). However, results of other previous studies are mixed: Aβ was sometimes related to episodic memory performance, and more consistently so in longitudinal studies (Hedden et al., 2013; Schultz et al., 2015; Clark et al., 2016, 2019; Farrell et al., 2017), but not always (Johnson et al., 2014; Oh et al., 2014; Song et al., 2015; Mielke et al., 2016), whereas a link with executive functions was demonstrated only in a few studies (Doherty et al., 2015; Clark et al., 2016; Mielke et al., 2016), in contrast to a majority of studies that did not find any significant link (Johnson et al., 2014; Hollands et al., 2015; Lim et al., 2015; Pietrzak et al., 2015; Song et al., 2015). In addition to VOI, inconsistencies could also be due to differences in the number of amyloid deposits across cohorts, with only studies including participants with the higher level of Aβ or tau pathology (i.e., Aβ/tau positive patients) showing a significant amyloid/tau-cognition association.

### Limitations

Beyond the choice of the region of interest, other methodological choices may have impacted our results. First, we selected volunteers devoid of many health and lifestyle factors (Baumgart et al., 2015; Kane et al., 2017; Livingston et al., 2017) that typically increase the risk for pathological cognitive decline (e.g., no smoking, limited caffeine and alcohol intake, relatively low body mass index, no current treatment for psychiatric disorders, etc.). This may decrease the variability in certain factors, especially concerning allostatic load measures. However, our selection bias allows assessing associations with cognition away from these risk factors.

Moreover, the lack of associations between AD biomarker and cognition may also be due to a low level of AD biomarkers. Indeed, all our participants had a negative biomarker status for AD pathology. Previous studies usually showed that when Aβ levels are low, the magnitude of the link between amyloid and cognition is small (Farrell et al., 2018; Leal et al., 2018). Consequently, a very large sample would be needed for identifying such effects in a cross-sectional study. The longitudinal follow-up of our participants should help to determine if the presence of sub-clinical level of AD biomarkers is associated with subsequent cognitive decline.

Finally, we could consider that our study had the potentially limited power for evaluating multiple risk and protective factors simultaneously, given the sample size and extensive phenotyping of our 101 participants.

### Conclusion

Our results show that domain-specific cognition in normal aging is determined by a combination of modifiable and non-modifiable factors, with a better cognitive reserve and, more precisely, crystallized intelligence being the best predictor for performance in all cognitive domains, as well as sex for episodic memory and age for attention. In addition, our cross-sectional study showed that domain-specific cognition was mostly unrelated to APOE genotype and AD biomarkers in the healthy late-middle-aged population. Future longitudinal studies focusing on the same population are required to assess further associations between domain-specific cognitive decline, PET, and genetic markers.

## Data Availability Statement

The raw data supporting the conclusions of this article will be made available by the authors, without undue reservation.

## Ethics Statement

The studies involving human participants were reviewed and approved by Ethics Committee of the Faculty of Medicine, University of Liège, Liège, Belgium. The patients/participants provided their written informed consent to participate in this study.

## Author Contributions

FC, CB, GV, ES, PM, CP, MV, and JN contributed to the conception and design of the study. JN, MV, DC, EK, GB, VM, CS, GV, CB, and FC participated in the collection of the data. JN performed the statistical analysis. JN, FC, CB, and GV contributed to the interpretation of the data and wrote the first draft of the manuscript. All authors participated in manuscript revision.

## Conflict of Interest

The authors declare that the research was conducted in the absence of any commercial or financial relationships that could be construed as a potential conflict of interest.

## Footnotes

1 https://imputation.sanger.ac.uk/

2 http://hmri.info
