# Supplementary material for: Positive Effect of Cognitive Reserve on Episodic Memory, Executive and Attentional Functions Taking Into Account Amyloid-Beta, Tau, and Apolipoprotein E Status
Source: Front Aging Neurosci. 2021 May 28;13:666181. doi: 10.3389/fnagi.2021.666181 (PMC8194490; doi:10.3389/fnagi.2021.666181)
Supplement: Supplementary file 1 [file Table_1.docx]

# Supplement 1

## Amyloid-beta PET scaling to Centiloid units

As our data contained Aβ-PET images obtained using two radiotracers, [18F]Flutemetamol (N = 97) and [18F]Florbetapir (N = 3), we have scaled their SUVR values to Centiloid units (CL) following guidelines suggested previously (1). First, in comparison to standard Centiloid PET SPM-based processing pipeline for quantitative Aβ plaque estimation (1), our processing pipeline had introduced a few modifications aiming to increase Aβ estimation quality (i.e., MRI segmentation based on hMRI toolbox, MRI normalization performed using DARTEL via hMRI), therefore, we have validated our pipeline using external data consisting in Pittsburg compound B (PiB) PET scans, and we have met validation requirements (linear regression parameters between results obtained by standardized pipeline and our modified pipeline: slope between .98 and 1.02, intercept between -2 and 2 CL, R2 > .98; see Figure S.1.A). Second, we have validated our pipeline for each radiotracer (Flutemetamol and Florbetapir) on external data (2,3), where main condition was to achieve R2 > .70 in linear regression between PiB PET and other respective PET tracer using our pipeline (see Figure S.1.B and Figure S.1.C). Third, scaling equations which allow direct conversion from Aβ-PET SUVR values to CL for standardized mask, which is available on the Global Alzheimer Association Interactive Network (GAAIN) website (http://www.gaain.org/centiloid-project), were calculated according to linear regressions applied for external data (2,3) using our modified pipeline. Thus the following linear conversion computations were applied for our own dataset in the present article: Flutemetamol Centiloid = 116 × Flutemetamol SUVR – 115; and Florbetapir Centiloid = 175 × Florbetapir SUVR – 183. In addition to that, several other brain in-lab masks (global, medial prefrontal cortex, medial temporal lobe, hippocampus, default mode network, separate masks for brain networks responsible for episodic memory, executive functions, and attention) were also used to convert Aβ-PET SUVR values to CL, but were used for analyses not presented in this article. The latter conversion was based on mean SUVR values for young controls and AD patients (1) calculated in the regions covered in those masks with our in-lab processing pipeline. Subsequently, new Centiloid scaling equations for Flutemetamol SUVR and Florbetapir SUVR values were computed for each specific mask.


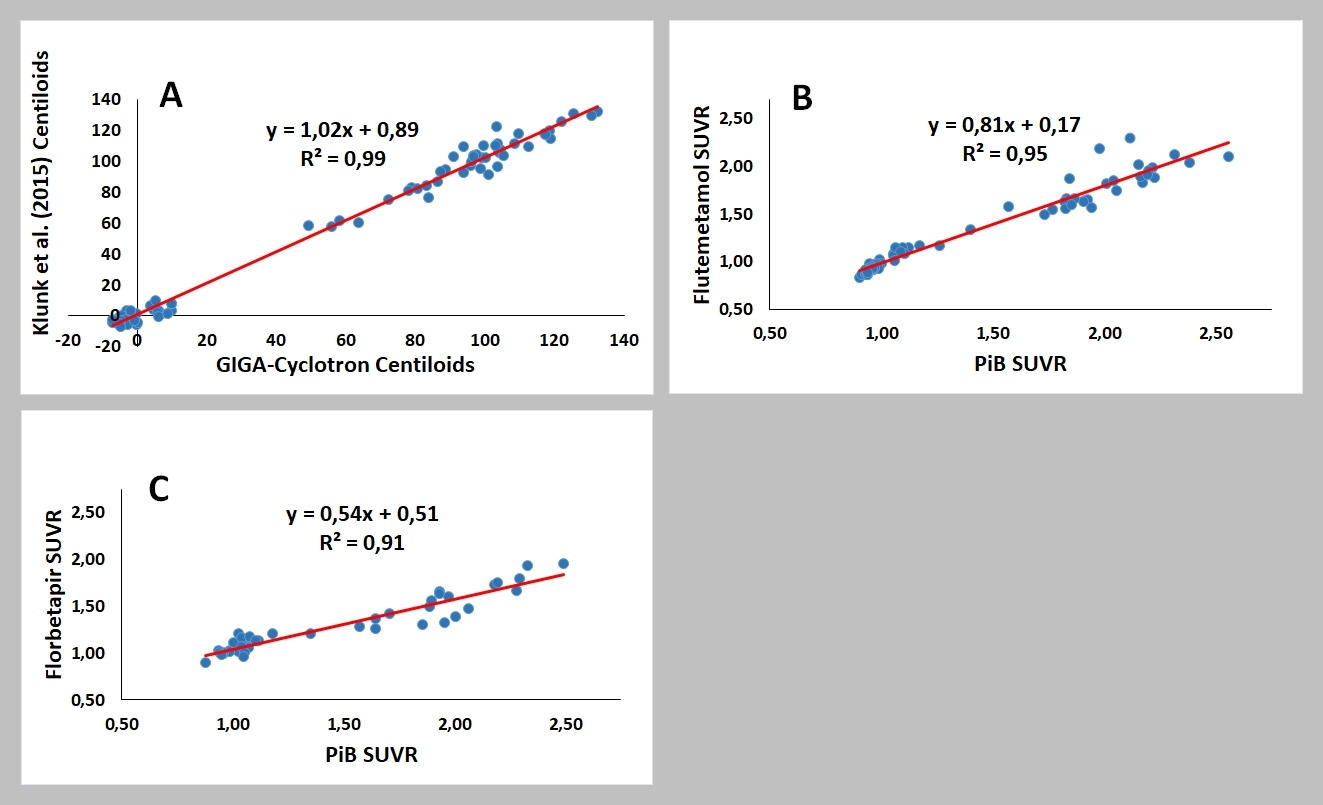


Figure S.1. **A (top left):** Scatter plot visualizing the link between CL values computed for the same sample of 78 PiB PET scans using two processing pipelines: Klunk et al. (2015) pipeline on Y axis, and our in-lab processing pipeline on X axis. **B (top right):** Scatter plot visualizing the link between SUVR values computed for the same sample of 74 individuals (3) using our in-lab processing pipeline for two radiotracers: PiB PET on X axis, and Flutemetamol PET on Y axis. **C (bottom left):** Scatter plot visualizing the link between SUVR values computed for the same sample of 45 individuals (2) using our in-lab processing pipeline for two radiotracers: PiB PET on X axis, and Florbetapir PET on Y axis.

**References**

1. Klunk WE, Koeppe RA, Price JC, Benzinger TL, Devous MD, Jagust WJ, et al. The Centiloid project: Standardizing quantitative amyloid plaque estimation by PET. Alzheimer’s Dement [Internet]. 2015;11(1):1–15.e4. Available from: http://dx.doi.org/10.1016/j.jalz.2014.07.003

2. Navitsky M, Joshi AD, Kennedy I, Klunk WE, Rowe CC, Wong DF, et al. Standardization of amyloid quantitation with florbetapir standardized uptake value ratios to the Centiloid scale. Alzheimer’s Dement [Internet]. 2018;14(12):1565–71. Available from: https://doi.org/10.1016/j.jalz.2018.06.1353

3. Battle MR, Pillay LC, Lowe VJ, Knopman D, Kemp B, Rowe CC, et al. Centiloid scaling for quantification of brain amyloid with [ 18 F]flutemetamol using multiple processing methods. EJNMMI Res. 2018;8.
