# Supplementary material for: Positive Effect of Cognitive Reserve on Episodic Memory, Executive and Attentional Functions Taking Into Account Amyloid-Beta, Tau, and Apolipoprotein E Status
Source: Front Aging Neurosci. 2021 May 28;13:666181. doi: 10.3389/fnagi.2021.666181 (PMC8194490; doi:10.3389/fnagi.2021.666181)
Supplement: Supplementary file 2 [file Table_2.docx]

# Appendix A

## Satistical models with amyloid-beta and tau outliers included

## Episodic Memory

### *Simultaneous Effect of Modifiable and Non-Modifiable Factors*

Table A.1. Statistical outcome of the GLMM examining the associations between episodic memory (dependent variable) and: **A:** scores of cognitive reserve and allostatic load, and Aβ (n = 100); **B:** scores of cognitive reserve and allostatic load, Aβ, Tau and APOE (n = 62).

| **Model A** (n = 100) | **Estimate ± SE** | **F value (df)** | **P** |
| --- | --- | --- | --- |
| Sex* | -.41 ± .21 | 3.76 (1,93) | .06 |
| Age | -.004 ± .10 | .0 (1,93) | .97 |
| fNART | .31 ± .10 | 10.14 (1,93) | **.002** (R_sp_^2^**=**.10) |
| Lipid metabolism | -.07 ± .10 | .50 (1,93) | .48 |
| Sympathetic functioning | -.10 ± .10 | 1.19 (1,93) | .28 |
| Aβ, Neocortex | -.1 ± .10 | .87 (1,93) | .35 |
|  |  |  |  |
| **Model B** (n = 62) | **Estimate ± SE** | **F value (df)** | **P** |
| Sex* | -.08 ± .28 | .07 (1,53) | .79 |
| Age | -.21 ± .13 | 2.45 (1,53) | .12 |
| fNART | .28 ± .13 | 4.43 (1,53) | **.04** (R_sp_^2^**=**.08) |
| Lipid metabolism | .02 ± .13 | .02 (1,53) | .90 |
| Sympathetic functioning | -.11 ± .14 | .68 (1,53) | .41 |
| Aβ, Neocortex | -.01 ± .15 | .01 (1,53) | .92 |
| THK5351 uptake, Braak I/II | -.05 ± .13 | .15 (1,53) | .70 |
| APOE | .13 ± .35 | .14 (1,53) | .71 |

SE, Standard Error; df, degrees of freedom; Rsp2, Semi-partial R2; fNART, National Adult Reading Test (French version); Aβ , amyloid βeta; APOE, Apolipoprotein E.

*Sex: 1 = male; 2 = female.

## Executive Functions

### *Simultaneous Effect of Modifiable and Non-Modifiable Factors*

Table A.2. Statistical outcome of the GLMM examining the associations between executive functions (dependent variable) and: **A:** scores of cognitive reserve and allostatic load, and Aβ (n = 100); **B:** scores of cognitive reserve and allostatic load, Aβ, Tau and APOE (n = 62).

| **Model A** (n = 100) | **Estimate ± SE** | **F value (df)** | **P** |
| --- | --- | --- | --- |
| Sex* | .07 ± .20 | .12 (1,93) | .73 |
| Age | -.18 ± .10 | 3.36 (1,93) | .07 |
| fNART | .46 ± .09 | 25.70 (1,93) | **< .0001** (R_sp_^2^**=**.22) |
| Lipid metabolism | .07 ± .09 | .55 (1,93) | .46 |
| Sympathetic functioning | .12 ± .09 | 1.77 (1,93) | .19 |
| Aβ, Neocortex | .04 ± .10 | .17 (1,93) | .68 |
|  |  |  |  |
| **Model B** (n = 62) | **Estimate ± SE** | **F value (df)** | **P** |
| Sex* | -.16 ± .24 | .46 (1,53) | .50 |
| Age | -.16 ± .12 | 1.91 (1,53) | .17 |
| fNART | .57 ± .12 | 24.81 (1,53) | **< .0001** (R_sp_^2^**=**.32) |
| Lipid metabolism | -.002 ± .12 | .0 (1,53) | .98 |
| Sympathetic functioning | .05 ± .12 | .15 (1,53) | .70 |
| Aβ, Neocortex | .11 ± .13 | .64 (1,53) | .43 |
| THK5351 uptake, Braak I/II | .24 ± .12 | 4.28 (1,53) | **.04** (R_sp_^2^**=**.07) |
| APOE | .46 ± .31 | 2.27 (1,53) | .14 |

SE, Standard Error; df, degrees of freedom; Rsp2, Semi-partial R2; fNART, National Adult Reading Test (French version); Aβ , amyloid βeta; APOE, Apolipoprotein E.

*Sex: 1 = male; 2 = female.

## Attentional Functioning

### *Simultaneous Effect of Modifiable and Non-Modifiable Factors*

Table A.3. Statistical outcome of the GLMM examining the associations between attentional functioning (dependent variable) and: **A:** scores of cognitive reserve and allostatic load, and Aβ (n = 100); **B:** scores of cognitive reserve and allostatic load, Aβ, Tau and APOE (n = 62).

| **Model A** (n = 100) | **Estimate ± SE** | **F value (df)** | **P** |
| --- | --- | --- | --- |
| Sex* | .03 ± .21 | .02 (1,93) | .89 |
| Age | -.25 ± .10 | 6.46 (1,93) | **.01** (R_sp_^2^**=**.06) |
| fNART | .37 ± .09 | 15.40 (1,93) | **.0002** (R_sp_^2^**=**.14) |
| Lipid metabolism | .02 ± .10 | .05 (1,93) | .83 |
| Sympathetic functioning | .16 ± .09 | 2.98 (1,93) | .09 |
| Aβ, Neocortex | -.03 ± .10 | .11 (1,93) | .74 |
|  |  |  |  |
| **Model B** (n = 62) | **Estimate ± SE** | **F value (df)** | **P** |
| Sex* | .08 ± .26 | .09 (1,53) | .76 |
| Age | -.23 ± .13 | 3.27 (1,53) | .08 |
| fNART | .44 ± .13 | 12.32 (1,53) | **.001** (R_sp_^2^**=**.19) |
| Lipid metabolism | -.03 ± .13 | .05 (1,53) | .82 |
| Sympathetic functioning | .13 ± .13 | .98 (1,53) | .33 |
| Aβ, Neocortex | .006 ± .15 | .0 (1,53) | .97 |
| THK5351 uptake, Braak I/II | .07 ± .13 | .29 (1,53) | .59 |
| APOE | .18 ± .33 | .29 (1,53) | .59 |

SE, Standard Error; df, degrees of freedom; Rsp2, Semi-partial R2; fNART, National Adult Reading Test (French version); Aβ , amyloid βeta; APOE, Apolipoprotein E.

*Sex: 1 = male; 2 = female.

# Appendix B

## Satistical models with more targeted VOIs of amyloid-beta (posterior cingulate cortex and precuneus) and tau (entorhinal cortex)

## Episodic Memory

### *Simultaneous Effect of Modifiable and Non-Modifiable Factors*

Table B.1. Statistical outcome of the GLMM examining the associations between episodic memory (dependent variable) and: **A:** scores of cognitive reserve and allostatic load, and Aβ (n = 100); **B:** scores of cognitive reserve and allostatic load, Aβ, Tau and APOE (n = 62).

| **Model A** (n = 100) | **Estimate ± SE** | **F value (df)** | **P** |
| --- | --- | --- | --- |
| Sex* | -.44 ± .21 | 4.44 (1,93) | .04 |
| Age | -.03 ± .10 | .06 (1,93) | .80 |
| fNART | .32 ± .10 | 10.91 (1,93) | **.001** (R_sp_^2^**=**.10) |
| Lipid metabolism | -.06 ± .10 | .43 (1,93) | .51 |
| Sympathetic functioning | -.11 ± .10 | 1.29 (1,93) | .26 |
| Aβ, PCC and precuneus | -.02 ± .10 | .05 (1,93) | .83 |
|  |  |  |  |
| **Model B** (n = 62) | **Estimate ± SE** | **F value (df)** | **P** |
| Sex* | -.07 ± .27 | .06 (1,53) | .81 |
| Age | -.23 ± .13 | 3.07 (1,53) | .09 |
| fNART | .28 ± .13 | 4.79 (1,53) | **.03** (R_sp_^2^**=**.08) |
| Lipid metabolism | .02 ± .13 | .02 (1,53) | .90 |
| Sympathetic functioning | -.13 ± .14 | .94 (1,53) | .34 |
| Aβ, PCC and precuneus | .10 ± .14 | .52 (1,53) | .47 |
| THK5351 uptake, entorhinal cortex | -.05 ± .13 | .13 (1,53) | .72 |
| APOE | .25 ± .34 | .52 (1,53) | .47 |

SE, Standard Error; df, degrees of freedom; Rsp2, Semi-partial R2; fNART, National Adult Reading Test (French version); Aβ , amyloid βeta; PCC, posterior cingulate cortex; APOE, Apolipoprotein E.

*Sex: 1 = male; 2 = female.

## Executive Functions

### *Simultaneous Effect of Modifiable and Non-Modifiable Factors*

Table B.2. Statistical outcome of the GLMM examining the associations between executive functions (dependent variable) and: **A:** scores of cognitive reserve and allostatic load, and Aβ (n = 100); **B:** scores of cognitive reserve and allostatic load, Aβ, Tau and APOE (n = 62).

| **Model A** (n = 100) | **Estimate ± SE** | **F value (df)** | **P** |
| --- | --- | --- | --- |
| Sex* | .08 ± .20 | .18 (1,93) | .68 |
| Age | -.17 ± .09 | 3.13 (1,93) | .08 |
| fNART | .46 ± .09 | 25.52 (1,93) | **< .0001** (R_sp_^2^**=**.22) |
| Lipid metabolism | .07 ± .09 | .51 (1,93) | .48 |
| Sympathetic functioning | .12 ± .09 | 1.82 (1,93) | .18 |
| Aβ, PCC and precuneus | .01 ± .09 | .02 (1,93) | .90 |
|  |  |  |  |
| **Model B** (n = 62) | **Estimate ± SE** | **F value (df)** | **P** |
| Sex* | -.06 ± .24 | .07 (1,53) | .80 |
| Age | -.15 ± .12 | 1.77 (1,53) | .19 |
| fNART | .55 ± .11 | 23.08 (1,53) | **< .0001** (R_sp_^2^**=**.30) |
| Lipid metabolism | -.03 ± .12 | .06 (1,53) | .81 |
| Sympathetic functioning | .04 ± .12 | .13 (1,53) | .72 |
| Aβ, PCC and precuneus | .16 ± .13 | 1.52 (1,53) | .22 |
| THK5351 uptake, entorhinal cortex | .18 ± .11 | 2.58 (1,53) | .11 |
| APOE | .53 ± .30 | 3.04 (1,53) | .09 |

SE, Standard Error; df, degrees of freedom; Rsp2, Semi-partial R2; fNART, National Adult Reading Test (French version); Aβ , amyloid βeta; PCC, posterior cingulate cortex; APOE, Apolipoprotein E.

*Sex: 1 = male; 2 = female.

## Attentional Functioning

### *Simultaneous Effect of Modifiable and Non-Modifiable Factors*

Table B.3. Statistical outcome of the GLMM examining the associations between attentional functioning (dependent variable) and: **A:** scores of cognitive reserve and allostatic load, and Aβ (n = 100); **B:** scores of cognitive reserve and allostatic load, Aβ, Tau and APOE (n = 62).

| **Model A** (n = 100) | **Estimate ± SE** | **F value (df)** | **P** |
| --- | --- | --- | --- |
| Sex* | .01 ± .20 | .0 (1,93) | .96 |
| Age | -.24 ± .10 | 6.32 (1,93) | **.01** (R_sp_^2^**=**.06) |
| fNART | .37 ± .09 | 15.98 (1,93) | **.0001** (R_sp_^2^**=**.15) |
| Lipid metabolism | .03 ± .09 | .07 (1,93) | .79 |
| Sympathetic functioning | .16 ± .09 | 3.07 (1,93) | .08 |
| Aβ, PCC and precuneus | -.07 ± .09 | .48 (1,93) | .49 |
|  |  |  |  |
| **Model B** (n = 62) | **Estimate ± SE** | **F value (df)** | **P** |
| Sex* | .09 ± .26 | .13 (1,53) | .72 |
| Age | -.23 ± .13 | 3.26 (1,53) | .08 |
| fNART | .44 ± .12 | 12.54 (1,53) | **.0008** (R_sp_^2^**=**.19) |
| Lipid metabolism | -.03 ± .13 | .07 (1,53) | .80 |
| Sympathetic functioning | .13 ± .13 | 1.01 (1,53) | .32 |
| Aβ, PCC and precuneus | .005 ± .14 | .0 (1,53) | .97 |
| THK5351 uptake, entorhinal cortex | .06 ± .12 | .26 (1,53) | .61 |
| APOE | .19 ± .32 | .33 (1,53) | .57 |

SE, Standard Error; df, degrees of freedom; Rsp2, Semi-partial R2; fNART, National Adult Reading Test (French version); Aβ , amyloid βeta; PCC, posterior cingulate cortex; APOE, Apolipoprotein E.

*Sex: 1 = male; 2 = female.
